# Assessment of active TGFβ with a bioassay

**DOI:** 10.1186/1546-0096-9-S1-P198

**Published:** 2011-09-14

**Authors:** Annemieke Laarhoven, Katja Heitink, Wilco de Jager, Berent Prakken, Femke van Wijk

**Affiliations:** 1 Department of Experimental Immunohaematology, Sanquin Research Amsterdam and Landsteiner Laboratory, Academic Medical Center, University of Amsterdam, Amsterdam, the Netherlands; 2 Department of Pediatric Immunology, Wilhelmina Children's Hospital, University Medical Center Utrecht (UMCU), Utrecht, the Netherlands

## Background

Transforming Growth Factor beta (TGFβ) is a highly pleiotropic cytokine that is involved in numerous signalling pathways, including immune homeostasis. It is secreted by all immune cell lineages and is classically viewed as an anti-inflammatory cytokine. TGFβ is associated with regulatory T cell (Treg) induction, and Treg-function. However, in combination with pro-inflammatory cytokines (IL-6, IL-β and IL-23) TGFβ will induce proinflammatory Th17 cells. In several autoimmune diseases, including Juvenile Idiopathic Arthritis (JIA), a disrupted Th17-Treg balance, lower Treg numbers or less functional Tregs have been described.

Unfortunately TGFβ production is hard to measure. Popular methods like flow cytometry or ELISA are not reliable, not sensitive or require activation of all latent TGFβ present, which might not be representative.

## Aim

To develop a reliable and sensitive test to assess active TGFβ level in serum, and T cell cultures.

## Methods

The mink cell line CCL-64 is used to assess TGFβ levels in serum. Cells are cultured in serum free medium for 24hrs before serum is added. After 19hrs incubation 3H-thymidine is added and cell proliferation is assessed after 5hrs.

## Evaluation

The highly sensitive cell line CCL-64 is currently established in our lab.The next months serum samples and T cell cultures of JIA and other autoimmune patients will be tested.

## Conclusion

A sensitive bioassay for active TGFβ may provide a better understanding of the role of TGFβ in Treg number and function as well as the Th17-Treg balance in autoimmune diseases.

